# Hysterectomy accelerates sarcopenia risk in US women and mouse models

**DOI:** 10.3389/fendo.2026.1859421

**Published:** 2026-07-14

**Authors:** Shuquan Wan, Cuiping Gong

**Affiliations:** Shandong Provincial Third Hospital, Cheeloo College of Medicine, Shandong University, Ji’nan, China

**Keywords:** ferroptosis, hysterectomy, SAMP8 mice, sarcopenia, women

## Abstract

**Background:**

Sarcopenia represents a clinical condition with particular prevalence among postmenopausal women. Hysterectomy is a common gynecological surgical procedure associated with various complications. However, the relationship between hysterectomy and sarcopenia remains poorly investigated. This study aimed to explore the association between hysterectomy and sarcopenia risk.

**Method:**

Cross-sectional data from the National Health and Nutrition Examination Survey (NHANES, 2001-2018) was utilized for analysis. Sarcopenia was defined using the Foundation for the National Institutes of Health (FNIH) criteria based on ALM/BMI < 0.512 in women. Multivariable logistic regression and propensity score matching were applied to assess the association between hysterectomy and sarcopenia. In parallel, a senescence-accelerated mouse model (SAMP8) was used to examine the effects of hysterectomy on muscle function and related molecular pathways, including markers of protein degradation and ferroptosis.

**Results:**

In the NHANES cohort, hysterectomy was associated with an increased risk of sarcopenia after adjustment for covariates (OR = 1.35; 95% CI: 1.00–1.82; *p* = 0.049). The association was stronger in women who had undergone both hysterectomy and oophorectomy (OR = 2.06; 95% CI: 1.45–2.93; *p* < 0.001). In SAMP8 mice, hysterectomy was associated with reduced grip strength, shorter endurance time, and decreased muscle fiber size. Molecular analyses suggested activation of the FOXO1–MuRF-1/Atrogin-1 pathway and changes consistent with ferroptosis-related signaling.

**Conclusion:**

Hysterectomy appears to be associated with an increased risk of sarcopenia in women, and this association is supported by findings from an experimental mouse model. These results suggest potential involvement of muscle protein degradation and ferroptosis-related pathways, although further studies are needed to clarify causality.

## Introduction

Sarcopenia is a progressive, systemic skeletal muscle disorder involving the accelerated loss of muscle mass, strength, and function (1). It has been increasingly recognized as an important contributor to adverse health outcomes in older adults, including falls, fractures, disability in daily activities, and increased mortality (2–5). With the accelerating pace of global population aging, the prevalence of sarcopenia is rising worldwide, particularly across Asia where demographic transitions are occurring most rapidly. Epidemiological studies have reported that the prevalence of sarcopenia varies across populations, with estimates ranging from approximately 7% to 12% in Asian cohorts (6). A 2014 epidemiological investigation revealed that sarcopenia affected 7.3% of China’s elderly population aged ≥65 years, demonstrating significant gender disparity with higher prevalence in males compared to females (7).

Hysterectomy is the second most common surgical procedure among women, surpassed only by cesarean delivery (8–10). In the United States, approximately 600,000 patients undergo hysterectomy annually (11). In the European Union, this figure exceeds 400,000 (12). Indications for surgery include both benign and malignant gynecological conditions, as well as pelvic disorders. Although generally considered safe, hysterectomy has been associated with a range of long-term health outcomes (13). In addition to these complications, evidence suggests that hysterectomy may increase the risk of cardiovascular disease, hyperlipidemia, osteoporosis, and other metabolic disorders (14–16). These findings raise the possibility that hysterectomy may also influence musculoskeletal health. However, the relationship between hysterectomy and sarcopenia has received relatively little attention, and existing evidence remains limited and inconsistent.

Given that sarcopenia is influenced by both hormonal and systemic metabolic factors, it is biologically plausible that gynecological surgery may contribute to its development (17). Nevertheless, whether hysterectomy itself is independently associated with sarcopenia risk remains unclear and requires further investigation. In this study, we aimed to assess the relationship between hysterectomy and the prevalence of sarcopenia in the US population. We obtained data from the National Health and Nutrition Examination Survey (NHANES) for secondary data analysis to determine the observational association. Furthermore, we established a senescence-accelerated mouse model to explore whether hysterectomy is associated with changes in muscle function and related molecular pathways. By combining epidemiological and experimental approaches, we sought to provide complementary evidence regarding this association.

## Materials and methods

### Study design and participants

**Figure 1:**
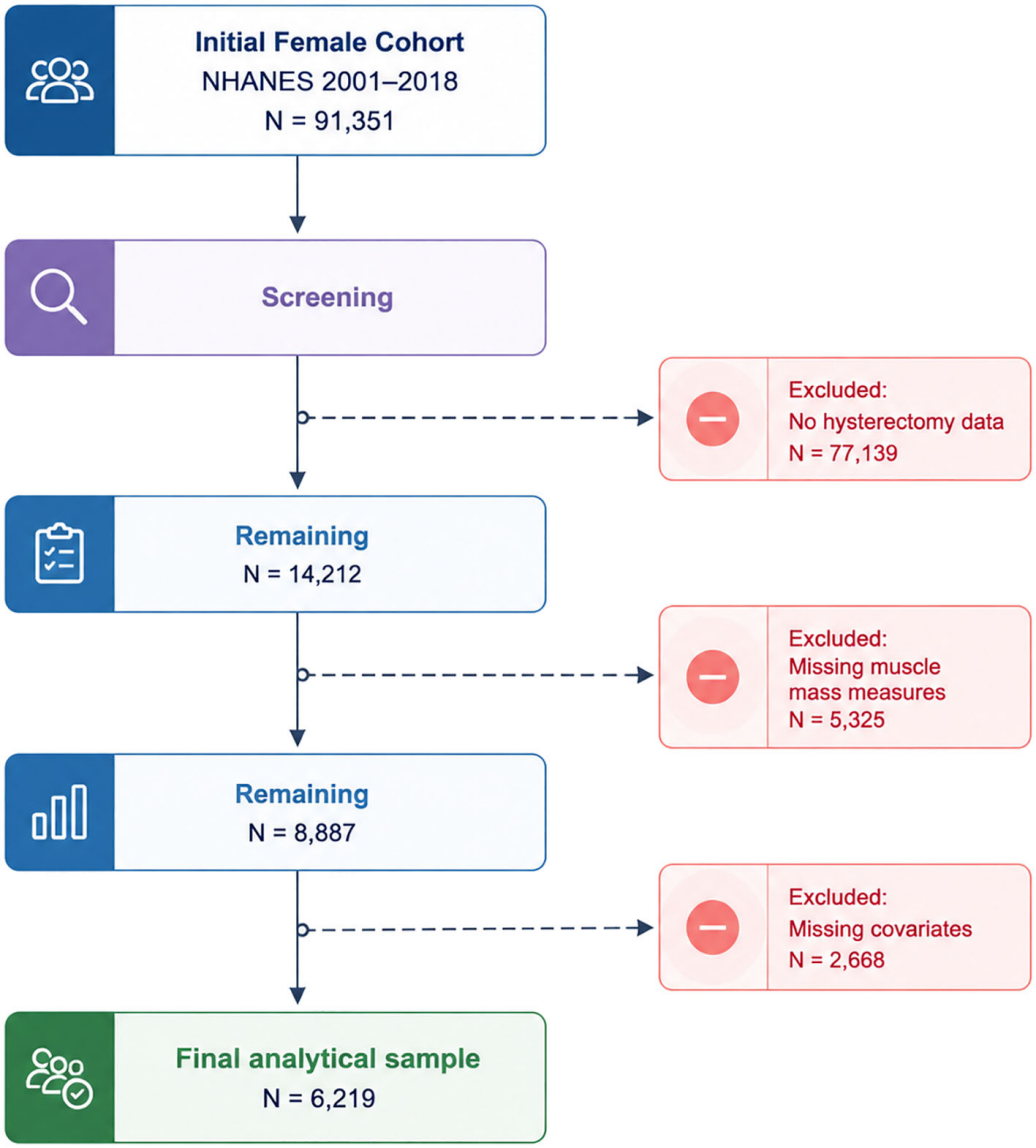
Flowchart of the participant selection from NHANES 2001–2018.

Our study population comprised all female respondents during the 2001–2018 cycle of the cross-sectional NHANES survey performed by the US National Center for Health Statistics (NCHS) (18). This survey employs a complex, multistage, probability-based approach to ensure the enrollment of a nationally representative sample of non-institutionalized civilians throughout the United States across all age groups. The exclusion criteria were as follows: (1) participants with incomplete surgical information (n=77139), (2) participants with missing muscle mass data (n=5325), (3) missing the required covariate information (n=2668). A total of 6,219 participants were enrolled after screening for further analysis. (**Figure 1**).

### Exposures and outcomes

Surgical status regarding both oophorectomy and hysterectomy was obtained from standardized self-report data collected via the NHANES Reproductive Health Questionnaire (RHQ series) during face-to-face interviews conducted at Mobile Examination Centers. Appendicular lean mass (ALM) and body mass index (BMI) were obtained from the Examination Data module of the database. The ALM/BMI ratio was calculated as the quotient of these values. Sarcopenia was diagnosed based on criteria recommended by the Foundation for the National Institutes of Health (FNIH), using sex-specific cutpoints of ALM/BMI < 0.512 for women (19).

### Covariates

Age, income-to-poverty ratio (PIR), race/ethnicity, educational attainment, and marital status were obtained directly from the demographic module. Dietary intake data, including daily protein (g/day) and total energy (kcal/day), were acquired from the 24-hour dietary recall component of the nutrition assessment module using automated multiple-pass methodology. Data regarding alcohol intake, tobacco use status, hypertension and diabetes diagnoses, and physical activity levels were obtained using standardized questionnaires administered by trained interviewers during face-to-face assessments. Tobacco use status was categorized based on standardized epidemiological criteria, with ‘current smoking’ defined as having consumed ≥100 cigarettes during the lifetime and currently smoking every day or some days. Alcohol consumption was defined as consuming ≥4 drinks per day for women. Physical activity status was defined as engaging in any moderate-intensity exercise, fitness, or recreational activities that cause slight increases in breathing or heart rate for at least 10 minutes per session on a weekly basis, including but not limited to brisk walking, cycling, swimming, or volleyball. Participants who self-reported a physician diagnosis of diabetes (affirmative response to the question: “Has a doctor ever told you that you have diabetes?”) were classified as having diabetes. Participants who self-reported a physician diagnosis of hypertension (affirmative response to the question: “Has a doctor ever told you that you have high blood pressure?”) were classified as having hypertension.

### Animals and groupings

Sixteen female SAMP8 mice and eight female SAMR1 mice (11 weeks old, Peking University Experimental Animal Center, Beijing, China) were used for the study. Animals were placed individually in cages maintained at 26 °C on a 12 hr./12 hr. light/dark cycle. After 7 days of adaptation feeding, the animals were randomly divided into 3 groups. The control (SAMR1+Sham) group was 8 SAMR-1 mice, the sarcopenia (SAMP8+Sham) group and the hysterectomy (SAMP8+Surg) group were both 8 SAMP-8 mice, both groups were given a conventional diet. Mice were euthanized at 40 weeks of age via cervical dislocation following deep anesthesia induced by 5% isoflurane (in room air) in an induction chamber connected to a calibrated vaporizer. Depth of anesthesia was confirmed by loss of pedal withdrawal reflex before cervical dislocation was performed. The right and left gastrocnemius (GAS) and tibialis anterior (TA) muscles were then harvested, weighed, and processed for subsequent analyses. All mouse manipulations were performed following the recommendations in the Guide for the Care and Use of Laboratory Animals published by the US National Institutes of Health (NIH publication no. 85–23 revised1996) and approved by the Ethics Committee and the Scientific Investigation Board of Qilu Hospital, Shandong University, China (DWLL-2024-042).

### Mouse hysterectomy protocol

The hysterectomy surgery was performed as previously described (20). Mice were anesthetized by intraperitoneal injection of 1% pentobarbital sodium (10 mL/kg). After the animal reached stable anesthesia, it was placed in the supine position. The abdominal skin was routinely disinfected, and a transverse incision approximately 0.5 cm in length was made in the lower midline abdomen. The uterus was exposed, and both bilateral uterine horns were carefully separated. The uterus was transected at the utero-ovarian junction and completely dissected, with meticulous care to avoid injuring the ovaries and adjacent uterine vasculature. Subsequently, at the junction of the cervical os and vagina, ligation was performed using 0–1 surgical suture. The uterus was excised proximal to the cervical ligation site. The abdominal cavity was closed, and the skin was sutured and disinfected.

### Muscle strength grip-strength test

To measure the muscle strength of the mice, we used the method of Hung-Wen Liu (21). A commercially available grip strength meter (Bioseb, Chaville, France) was used for our test. Each mouse was placed on the grid and then its tail was pulled with increasing force until it was unable to grasp the grid. The maximum force applied (N) was recorded. It was repeated three times and the average reading was calculated.

### Cross-sectional areas measurement

The right gastrocnemius muscle of mice was taken, sections were made from the middle part with the largest cross-sectional area, HE stained and observed under 200 magnification, 10 fields of view were randomly taken, and CSA was calculated by ImageJ software (NIH, Frederick, MD).

### RNA extraction and quantitative real-time PCR

Total RNA from gastrocnemius was extracted using TRIzol reagent method according to the kit manual (Invitrogen). 5 μg of RNA was used for reverse transcription. Briefly, RNAs were reverse-transcribed using the HiScript III 1st Strand cDNA Synthesis Kit (Vazyme), and random hexamers were used as primers. The RT reaction was set as follows: 25 °C for 5 minutes; 37 °C for 45 minutes; 85 °C for 5 minutes. Real-time PCR was performed using SYBR Green II (Vazyme) in the iQ5 system (BioRad) with the primers described in supplemental **Supplementary Table 1** at 95 °C for 5 minutes followed 40 cycles of 95 °C for 15 seconds and 60 °C for 45 seconds. The analysis method is ΔΔCt. Primer sequences are listed in **Supplementary Table 1**.

### Western blotting

Total protein was extracted from gastrocnemius in lysis buffer containing protease/phosphatase inhibitors. Protein was quantified using BCA method (Boster, AR0146) and separated on sodium dodecyl sulfate-polyacrylamide electrophoresis gels before being transferred to nitrocellulose membranes (Millipore). After being blocked for 1.5 hours in 5% non-fat milk, the bands were incubated overnight at 4 °C with primary antibodies. After washing, secondary antibodies were incubated for 1.5 hours. The bands were scanned and detected by a chemiluminescence instrument (General Electric Company, AI800RGB). The relative intensity of immunoreactive bands was assessed by Image J software. The results were normalized to β-Actin levels and expressed as % of control. All experiments were repeated at least thrice. Antibodies against Beta Actin (ab8226), 4-HNE (ab46545) were from Abcam (UK).

### Statistical analysis

Normal Q-Q plots were utilized to assess the distribution of the data. Continuous variables were analyzed using either Welch two-sample t tests or nonparametric Mann–Whitney U tests. Categorical variables were compared using either chi-square tests or Fisher’s exact tests.

Previous studies have demonstrated the effectiveness of propensity score matching (PSM) in reducing selection bias in retrospective studies (22–24). In our study, we employed PSM with the 1:3 nearest neighbor matching algorithm to match subjects.

Baseline characteristics were compared using independent samples t-tests for continuous variables and chi-square tests for categorical variables. The association between hysterectomy status and sarcopenia incidence was evaluated through univariate and multivariate logistic regression analyses, while the relationships between surgical exposure and continuous outcomes (ALM and ALM/BMI ratio) were assessed using univariate and multivariate linear regression models.

Quantitative data from animal experiments are presented as the mean ± SD. Differences between two or more groups were compared by two-way ANOVA and unpaired Student’s t-test, respectively. Significant differences were set as *p* < 0.05.

Data extraction, merging, statistical analyses, and figures were conducted using R (version 4.1.1) and SPSS (version 22).

## Results

### Participant characteristics before matching

A total of 6219 female subjects were included in this study after screening. Non-surgery group (n = 4838) had a mean age of 42.49 ± 14.91 years, while surgery group (n = 1381) had a mean age of 56.51 ± 12.11 years. **Table 1** presents the comparisons of baseline characteristics between the two groups. The results revealed statistically significant differences in the vast majority of characteristics, including age and education level (*p <* 0.001). This indicated the presence of significant selection bias between the two groups of subjects. Consequently, PSM was conducted to mitigate this phenomenon.

**Table 1:** Baseline characteristics for the subjects before matching.

Variables	Non-surgery (n = 4838)	Surgery (n = 1381)	P
Age, Mean ± SD	42.49 ± 14.91	56.51 ± 12.11	< 0.001
PIR, Mean ± SD	2.62 ± 1.66	2.81 ± 1.60	< 0.001
BMI, Mean ± SD	29.30 ± 7.79	30.28 ± 7.21	< 0.001
Energy intake, Mean ± SD	1836.74 ± 659.97	1661.76 ± 587.97	< 0.001
Protein intake, Mean ± SD	70.58 ± 28.41	63.55 ± 25.15	< 0.001
Race, n (%)			< 0.001
Mexican American	705 (14.6)	168 (11.7)	
Other Hispanic	396 (8.2)	75 (5.2)	
Non-Hispanic White	2165 (44.7)	769 (53.4)	
Non-Hispanic Black	997 (20.6)	348 (24.1)	
Other Race	575 (11.9)	81 (5.6)	
Education level, n (%)			< 0.001
Less than 9th grade	253 (5.2)	109 (7.6)	
9 - 11th grade	507 (10.5)	192 (13.3)	
High school graduate	993 (20.5)	386 (26.8)	
Some college or AA degree	1676 (34.6)	505 (35.0)	
College graduate or above	1409 (29.1)	249 (17.3)	
Alcohol consumption status, n (%)			0.151
Negative	4427 (91.5)	1301 (90.3)	
Positive	411 (8.5)	140 (9.7)	
Tobacco use status, n (%)			< 0.001
Negative	2910 (60.1)	716 (49.7)	
Positive	1928 (39.9)	725 (50.3)	
Hypertension, n (%)			< 0.001
Negative	3636 (75.2)	702 (48.7)	
Positive	1202 (24.8)	739 (51.3)	
Diabetes, n (%)			
Negative	4482 (92.7)	1226 (85.1)	
Positive	353 (7.3)	215 (14.9)	
Physical Activity, n (%)			0.092
Negative	2463 (50.9)	770 (53.4)	
Positive	2375 (49.1)	671 (46.6)	
Marital status, n (%)			< 0.001
Married	2161 (44.7)	759 (52.7)	
Widowed	283 (5.8)	200 (13.9)	
Divorced	550 (11.4)	271 (18.8)	
Separated	203 (4.2)	65 (4.5)	
Never married	1147 (23.7)	94 (6.5)	
Living with partner	494 (10.2)	52 (3.6)	

PIR, Poverty income ratio.

BMI, Body mass index.

SD, standard deviation.

P values were calculated by Welch two-sample t test or the Pearson chi-square test with Yates’ continuity.

### Construction of matched datasets

Following 1:3 propensity score matching, 804 women with hysterectomy were matched to 2,412 women without hysterectomy, resulting in a matched cohort of 3,216 participants. Analysis of baseline characteristics revealed the absence of selection bias (P > 0.05). Further details can be found in **Table 2**.

**Table 2:** Baseline characteristics for the subjects after matching.

Variables	Non-surgery (n = 2412)	Surgery (n = 804)	P
Age, Mean ± SD	53.31 ± 13.12	52.66 ± 11.45	0.183
PIR, Mean ± SD	2.84 ± 1.63	2.80 ± 1.55	0.436
BMI, Mean ± SD	30.95 ± 7.33	31.12 ± 7.48	0.566
Energy intake, Mean ± SD	1729.81 ± 587.81	1717.23 ± 612.91	0.662
Protein intake, Mean ± SD	66.35 ± 25.67	65.96 ± 26.13	0.712
Race, n (%)			0.235
Mexican American	347 (14.4)	131 (16.3)	
Other Hispanic	161 (6.7)	55 (6.8)	
Non-Hispanic White	1226 (50.8)	397 (49.4)	
Non-Hispanic Black	528 (21.9)	158 (19.7)	
Other Race	150 (6.2)	63 (7.8)	
Education level, n (%)			< 0.001
Less than 9th grade	191 (7.9)	78 (9.7)	
9 - 11th grade	272 (11.3)	113 (14.1)	
High school graduate	532 (22.1)	202 (25.1)	
Some college or AA degree	771 (32.0)	277 (34.5)	
College graduate or above	646 (26.8)	134 (16.7)	
Alcohol consumption status, n (%)			0.636
Negative	2209 (91.6)	732 (91.0)	
Positive	203 (8.4)	72 (9.0)	
Tobacco use status, n (%)			0.513
Negative	1309 (54.3)	447 (55.6)	
Positive	1103 (45.7)	357 (44.4)	
Hypertension, n (%)			0.803
Negative	1443 (59.8)	447 (59.3)	
Positive	969 (40.2)	327 (40.7)	
Diabetes, n (%)			0.619
Negative	2122 (88.0)	702 (87.3)	
Positive	290 (12.0)	102 (12.7)	
Physical Activity, n (%)			0.870
Negative	1262 (52.3)	418 (52.0)	
Positive	1150 (47.7)	386 (48.0)	
Marital status, n (%)			0.054
Married	1323 (54.9)	445 (55.3)	
Widowed	282 (11.7)	91 (11.3)	
Divorced	408 (16.9)	112 (13.9)	
Separated	119 (4.9)	33 (4.1)	
Never married	187 (7.8)	82 (10.2)	
Living with partner	93 (3.9)	41 (5.1)	

PIR: Poverty income ratio.

BMI: Body mass index

SD: standard deviation.

P values were calculated by Welch two-sample t test or the Pearson chi-square test with Yates’ continuity.

### Association between hysterectomy and sarcopenia

After propensity score matching, our analysis revealed a significant association between hysterectomy and adverse musculoskeletal outcomes. Compared to matched non-surgical controls, women undergoing hysterectomy exhibited lower appendicular lean mass, reduced ALM/BMI ratio, and a markedly higher incidence of sarcopenia (**Table 3**). After propensity score matching to balance potential confounders including Race, Education level, Age, PIR, Marital status, BMI, Energy intake, Protein intake, Alcohol consumption status, Tobacco use status, Hypertension, Diabetes, Physical activity, the association between hysterectomy and sarcopenia risk remained statistically significant (adjusted OR: 1.85, 95% confidence interval: 1.50-2.27, *p <* 0.001) (**Table 4**).

**Table 3:** Comparison of sarcopenia risk between the two groups.

Variables	Non-surgery (n = 2412)	Surgery (n = 804)	P
ALM, Mean ± SD	18.23 ± 4.42	17.34 ± 4.46	< 0.001
ALM/BMI, Mean ± SD	0.60 ± 0.11	0.57 ± 0.11	< 0.001
Sarcopenia, n (%)			< 0.001
Negative	1992 (82.6)	585 (72.8)	
Positive	420 (17.4)	219 (27.2)	

ALM: Appendicular lean mass.

ALM/BMI: Appendicular lean mass adjusted by body mass index.

SD: standard deviation.

P values were calculated by Welch two-sample t test or the Pearson chi-square test with Yates’ continuity.

**Table 4:** Associations of the surgery with muscle mass and sarcopenia.

Variables	Model 1		Model 2		Model 3	
	OR (95% CI)	P	OR (95% CI)	P	OR (95% CI)	P
ALM	-0.09 (-1.24 - -0.54)	< 0.001	-0.09 (-1.23 - -0.63)	< 0.001	-0.09 (-1.21 - -0.71)	< 0.001
ALM/BMI	-0.12 (-0.04 - -0.02)	< 0.001	-0.12 (-0.04 - -0.02)	< 0.001	-0.12 (-0.04 - -0.02)	< 0.001
Sarcopenia	1.78 (1.47 - 2.14)	< 0.001	1.84 (1.51 - 2.23)	< 0.001	1.85 (1.50 - 2.27)	< 0.001

OR, Odds ratio; CI, Confidence interval.

ALM, Appendicular lean mass.

ALM/BMI, Appendicular lean mass adjusted by body mass index.

Model1: Crude.

Model2: Adjust: Age, Race, Education level, PIR, Marital status.

Model3: Adjust: Race, Education level, Age, PIR, Marital status, BMI, Energy intake, Protein intake, Alcohol consumption status, Tobacco use status, Hypertension, Diabetes, Physical activity.

### Impact of different surgical approaches on sarcopenia

Our analysis confirms oophorectomy as an independent risk factor for sarcopenia. To delineate the specific contribution of hysterectomy independent of ovarian removal, we conducted a comparative analysis of different surgical approaches. Notably, while the combined hysterectomy-oophorectomy procedure showed the strongest association with sarcopenia risk (OR = 2.06, 95% CI: 1.60-2.65), hysterectomy alone maintained a significant independent association with increased sarcopenia incidence (OR = 1.35, 95% CI: 1.00-1.82), suggesting that uterine removal itself contributes to musculoskeletal deterioration beyond the effects of ovarian excision (**Table 5**).

**Table 5:** Associations of the surgery with muscle mass and sarcopenia.

Variables	Model 3	
	OR (95% CI)	P
ALM		
Hysterectomy	-0.62 (-0.95 - -0.28)	< 0.001
Hysterectomy+Oophorectomy	-1.00 (-1.31 - -0.68)	< 0.001
ALM/BMI		
Hysterectomy	-0.02 (-0.03 - -0.01)	< 0.001
Hysterectomy+Oophorectomy	-0.03 (-0.04 - -0.02)	< 0.001
Sarcopenia		
Hysterectomy	1.35 (1.00 - 1.82)	0.048
Hysterectomy+Oophorectomy	2.06 (1.60 - 2.65)	< 0.001

OR, Odds ratio; CI, Confidence interval.

Model3: Adjust: Race, Education level, Age, PIR, Marital status, BMI, Energy intake, Protein intake, Alcohol consumption status, Tobacco use status, Hypertension, Diabetes, Physical activity.

Non-surgery was set as reference.

### Hysterectomy alone exacerbates sarcopenia in SAMP8 mice

**Figure 2:**
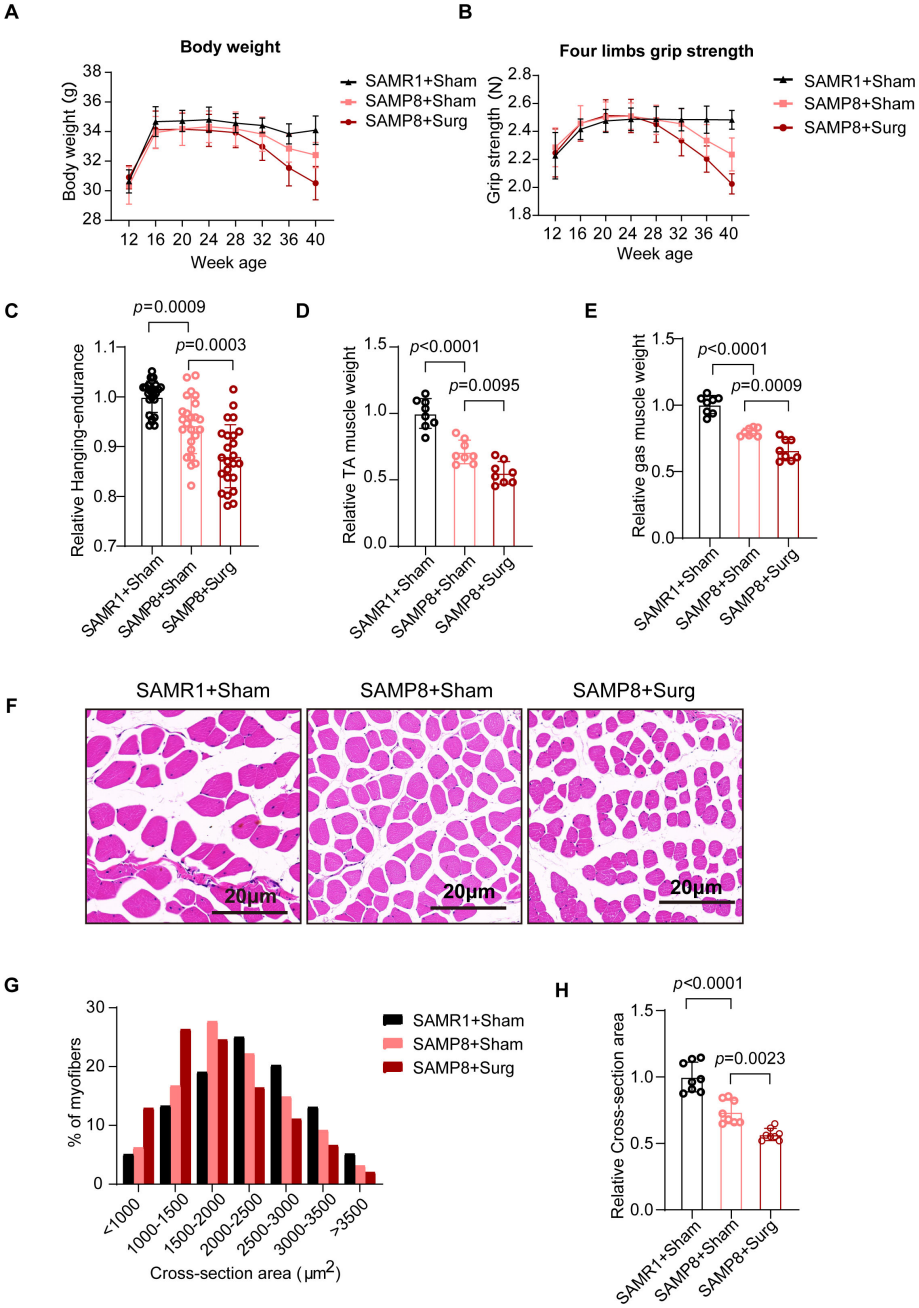
Hysterectomy exacerbates sarcopenia in mice. Eight 11-week-old SAMR1 mice were designated as the SAMR1+Sham group, while sixteen 11-week-old SAMP8 mice were equally divided into the SAMP8+Sham group and SAMP8+Surg group, undergoing sham surgery and hysterectomy respectively. **(A)** Changes in mice body weight over time (n=8). **(B)** Changes in mice grip strength over time (n=8). **(C)** Relative mice hanging endurance status at 40 weeks of age (n=24). **(D)** Relative mice TA muscle weight at 40 weeks of age (n=8). **(E)** Relative mice GAS muscle weight at 40 weeks of age (n=8). **(F)** Representative hematoxylin and eosin (HE)-stained cross-sections of gastrocnemius muscle tissue from the three experimental groups. **(G)** Distribution of myofiber cross-sectional area in the three groups. **(H)** Mean Myofiber Cross-Sectional Area in Gastrocnemius Muscle (n=8). The data were presented as the means ± SEM. Two-way ANOVA followed by the Bonferroni’s multiple comparisons test was used for statistical comparisons.

To investigate the impact of hysterectomy on sarcopenia, we conducted a study using a mouse model. Eight 12-week-old SAMP8 mice and eight SAMR1 mice underwent sham surgery, while another eight SAMP8 mice underwent hysterectomy. We observed that starting from week 28, both body weight and limb grip strength declined in SAMP8 mice, with a more pronounced decrease in the SAMP8+Surg group (**Figure 2A**, 2B). At week 40, the hanging time of SAMP8 mice was shorter than that of SAMR1 mice, and this difference was more significant in the SAMP8+Surg group (**Figure 2C**). After tissue collection, the wet weights of the gastrocnemius and tibialis anterior muscles in SAMP8 mice were found to be lower than those in SAMR1 mice, with the SAMP8+Surg group showing even greater reductions (**Figures 2D, E**). Hematoxylin and eosin (HE) staining of gastrocnemius muscle sections and subsequent analysis of myofiber cross-sectional area (CSA) revealed that SAMP8 mice had a smaller average myofiber CSA and a higher proportion of fibers with small CSA, with these changes being more evident in the SAMP8+Surg group (**Figures 2G, H**).

### Hysterectomy induces muscle atrophy and ferroptosis-related molecular signatures

**Figure 3:**
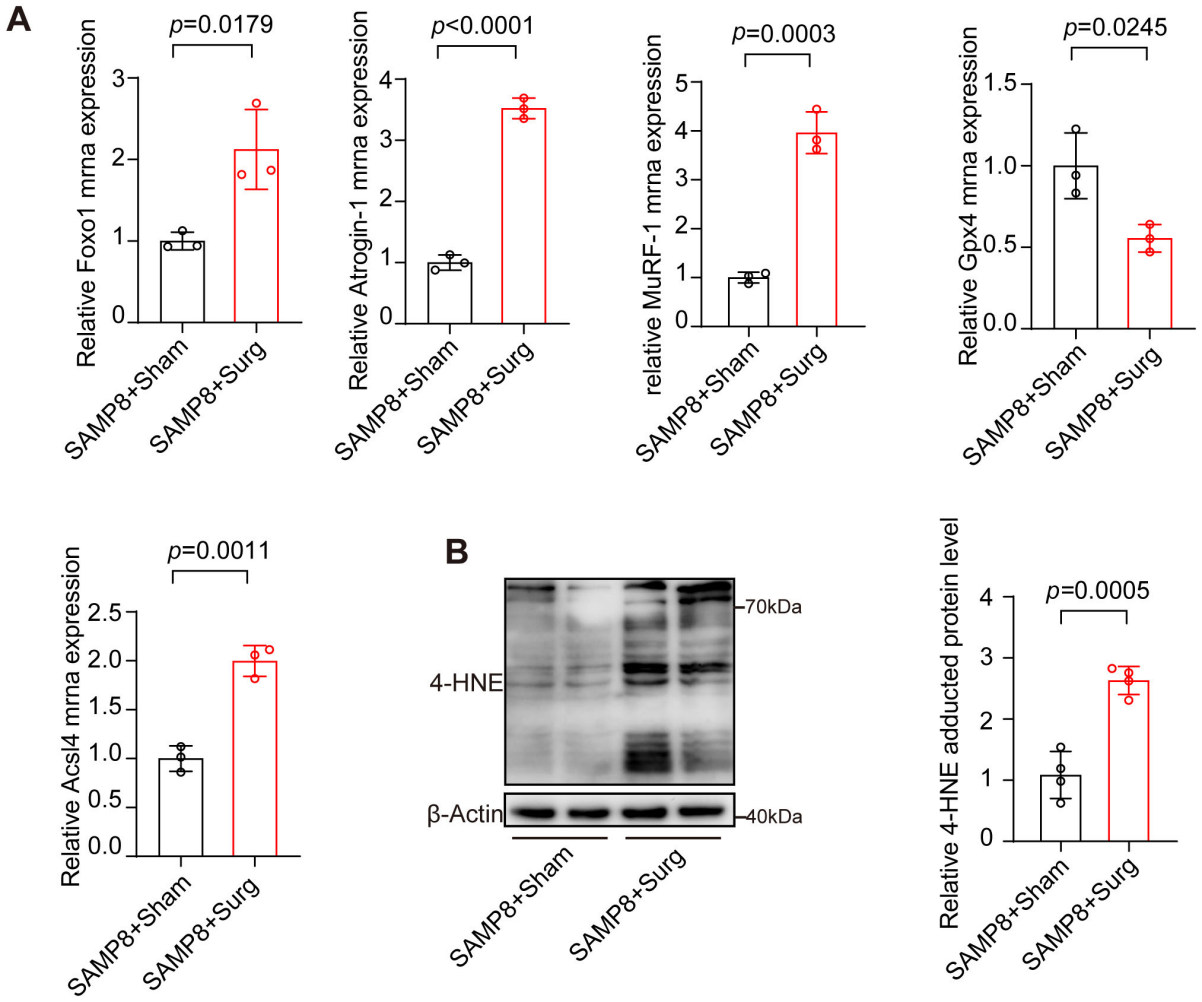
Hysterectomy activates the FOXO1/atrogin-1/MuRF-1 ubiquitin-proteasome pathway and ferroptotic signaling in skeletal muscle of SAMP8 mice. **(A)** Quantitative RT-qPCR analysis of Foxo1, Murf-1, Atrogin-1, Gpx4 and Acsl4 mRNA expression in the gastrocnemius muscle of sham-operated (SAMP8+Sham) and hysterectomized (SAMP8+Surg) SAMP8 mice (n = 3 per group). Expression levels were normalized to β-Actin and are presented relative to the sham group. **(B)** Representative Western blot images and quantitative analysis of 4-hydroxynonenal (4-HNE)-modified proteins in gastrocnemius muscle. Densitometric analysis was performed on the predominant immunoreactive region shown in the representative blot. Quantified values were normalized to β-actin. (n = 4 per group). All data are presented as mean ± SEM. *p* values were calculated using unpaired two-tailed Student’s t-test.

To investigate the mechanisms underlying the accelerated sarcopenia phenotype, we examined the expression of key regulators of the ubiquitin-proteasome and ferroptosis pathways. Hysterectomy significantly upregulated the mRNA expression of the transcription factor FOXO1 and its downstream E3 ubiquitin ligases MuRF-1 and Atrogin-1, compared to sham-operated controls. For ferroptosis-related signaling, GPX4 mRNA was significantly downregulated in hysterectomized mice, while ACSL4 mRNA was significantly upregulated (**Figure 3A**). Western blot analysis showed that 4-HNE-modified proteins was significantly increased in the SAM8+Surg group (**Figure 3B**). Collectively, these molecular data demonstrate that hysterectomy activates both the FOXO1-mediated ubiquitin-proteasome pathway and ferroptotic signaling in skeletal muscle.

## Discussion

This study integrates population-based epidemiological evidence with experimental animal data to explore the relationship between hysterectomy and sarcopenia. Overall, our findings suggest that hysterectomy is associated with an increased risk of sarcopenia, and this association appears to be independent of oophorectomy. In the NHANES cohort, women with hysterectomy showed a higher prevalence of sarcopenia after adjustment for major confounders. These observations were further supported by findings from the SAMP8 mouse model, in which hysterectomy was associated with accelerated declines in muscle mass and function.

At the mechanistic level, we observed activation of pathways involved in muscle protein degradation and oxidative stress regulation, including the FOXO1-mediated ubiquitin–proteasome system and ferroptosis-related signaling. Although causality cannot be fully established in the clinical dataset, the consistency between human and animal findings strengthens the biological plausibility of the association.

A key finding of our research is that even after accounting for the effects of ovarian removal, hysterectomy alone remained significantly associated with sarcopenia risk. Compared to young women, postmenopausal women exhibit reductions in physical strength, activity levels, and muscle mass (25, 26). Experimental studies have demonstrated that ovariectomized rats exhibit reductions in both muscle mass and muscle strength (27). This suggests that oophorectomy may be an independent risk factor for the development of sarcopenia. In our analysis, hysterectomy alone was associated with a modest but significant increase in sarcopenia risk, while combined hysterectomy and oophorectomy showed a stronger association. This pattern suggests a potential additive effect of ovarian removal on musculoskeletal outcomes. The observed gradient of risk across surgical groups may indicate a differential impact of reproductive organ removal on musculoskeletal health. To validate the causality of this clinical observation and explore underlying mechanisms, we performed hysterectomy in SAMP8 senescence-accelerated mice. The experimental results closely mirrored the clinical findings: compared to sham-operated controls, hysterectomized mice exhibited more severe reductions in limb grip strength, shorter hanging duration, lower gastrocnemius and tibialis anterior muscle wet weights, and more pronounced decreases in myofiber cross-sectional area. This animal model successfully recapitulated the clinical phenotype, providing compelling causal evidence for the association between hysterectomy and exacerbated sarcopenia.

To explore potential mechanisms, we examined markers of the ubiquitin–proteasome pathway. In skeletal muscle from hysterectomized mice, FOXO1 expression was increased along with its downstream targets MuRF-1 and Atrogin-1. These molecules are known regulators of protein degradation in skeletal muscle and have been implicated in a variety of muscle wasting conditions (28, 29). The upregulation of these atrogenes in our model suggests that hysterectomy accelerates muscle protein breakdown through this canonical pathway.

We also observed molecular changes consistent with ferroptosis-related processes. Specifically, GPX4 expression was reduced, whereas ACSL4 expression was increased in skeletal muscle following hysterectomy. In addition, elevated levels of 4-HNE were detected, indicating increased lipid peroxidation. These changes are consistent with previous reports linking ferroptosis to muscle degeneration and aging-related myopathy (30, 31). While our data do not directly assess iron accumulation or mitochondrial dysfunction, they suggest that ferroptosis-related pathways may be involved in the observed muscle changes.

Hormonal alterations may represent one potential explanation for these findings. Even when ovaries are preserved, hysterectomy may influence ovarian function and alter endocrine signaling through disruption of local vascular and feedback mechanisms. Although estrogen levels were not measured in this study, previous research has suggested that estrogen deficiency is associated with reduced muscle mass and strength (32–35). Through its receptors, which are widely distributed in skeletal muscle cells (36), estrogen mitigates inflammation and oxidative stress, thereby preventing muscle damage and facilitating muscle regeneration (37). Whether estrogen decline directly activates the FOXO1/atrogin-1/MuRF-1 axis or facilitates ferroptosis in skeletal muscle remains an open question that warrants further investigation.

Iron overload, as noted in our original hypothesis, may serve as a bridge linking hormonal changes to ferroptosis. Studies have indicated that postmenopausal women experience increased iron accumulation (38, 39), and this may also occur in women following hysterectomy. The vulnerability of skeletal muscle to iron dysregulation stems from its role as the organism’s primary iron reservoir (40). When iron storage capacity is exceeded, subsequent iron-mediated oxidative stress and inflammatory activation create a pathological microenvironment that disrupts the delicate balance of muscle protein synthesis and degradation (41). Our finding of increased 4-HNE supports the notion that iron overload may indeed occur in this context. Iron overload similarly predisposes to ferroptosis activation, establishing this iron-dependent cell death pathway as a crucial mechanistic component in sarcopenia pathogenesis (42, 43).

The primary strength of this study lies in the combination of large-scale population-based observational research with controlled animal experiments, thereby providing evidence for causality alongside association. In the clinical analysis, we employed propensity score matching and multivariable adjustment to minimize confounding bias. The animal experiment utilized a recognized senescence-accelerated model, enabling the simulation of human age-related sarcopenia progression within a relatively short observation period. Furthermore, the molecular analyses directly demonstrate activation of specific atrophy and ferroptosis pathways, providing mechanistic depth that is often lacking in translational studies.

However, several limitations should be acknowledged. First, the cross-sectional design of the clinical component limits definitive inference regarding causal temporality, despite supplementation by animal experiments. Second, our animal experiment included only a hysterectomy group (without oophorectomy) and a sham group. While this directly models the “hysterectomy alone” condition, it does not allow us to test the additive effect of oophorectomy. Future rodent studies with additional surgical groups are needed to fully recapitulate the clinical stratification. Third, we used the SAMP8 accelerated aging mouse model, which is well-established for sarcopenia research (44). However, as noted in systematic comparisons of aging models, SAMP8 mice develop multiple age-related pathologies that could influence some of the molecular changes we observed (45–47). Future studies using non-accelerated aging models with selective uterine removal would help isolate the specific uterine contribution to sarcopenia. Fourth, serum hormone levels were not measured due to limited sample availability and prioritization of tissue for molecular analyses. Finally, it should be acknowledged that because our study relied on the NHANES database, several clinically relevant variables, including surgical indication, menopausal status, and muscle strength, were not available. As a result, our diagnostic approach does not fully align with the EWGSOP2 criteria for sarcopenia, and the generalizability of our findings to other populations should be interpreted with caution.

The findings of this study carry important clinical and public health implications. They suggest that gynecologists, when considering hysterectomy, should carefully weigh the absolute necessity of the procedure against its potential long-term risks to musculoskeletal health. For women who have undergone the surgery, incorporating resistance training, adequate protein intake, and regular body composition monitoring into their long-term postoperative health management plan is strongly recommended.

## Conclusion

In conclusion, our study suggests that hysterectomy is associated with an increased risk of sarcopenia, independent of oophorectomy. Experimental findings in mice further support a potential link between uterine removal and accelerated muscle decline. Mechanistically, pathways related to muscle protein degradation and ferroptosis may be involved, although further studies are needed to clarify causal relationships and underlying biological processes.

## Data Availability

The original contributions presented in the study are included in the article/**Supplementary Material**. Further inquiries can be directed to the corresponding author.
