# Supplementary figures and images for: Hysterectomy accelerates sarcopenia risk in US women and mouse models
Source: Front Endocrinol (Lausanne). 2026 Jul 14;17:1859421. doi: 10.3389/fendo.2026.1859421 (PMC13407118; doi:10.3389/fendo.2026.1859421)

Fig.3 B

4-HNE

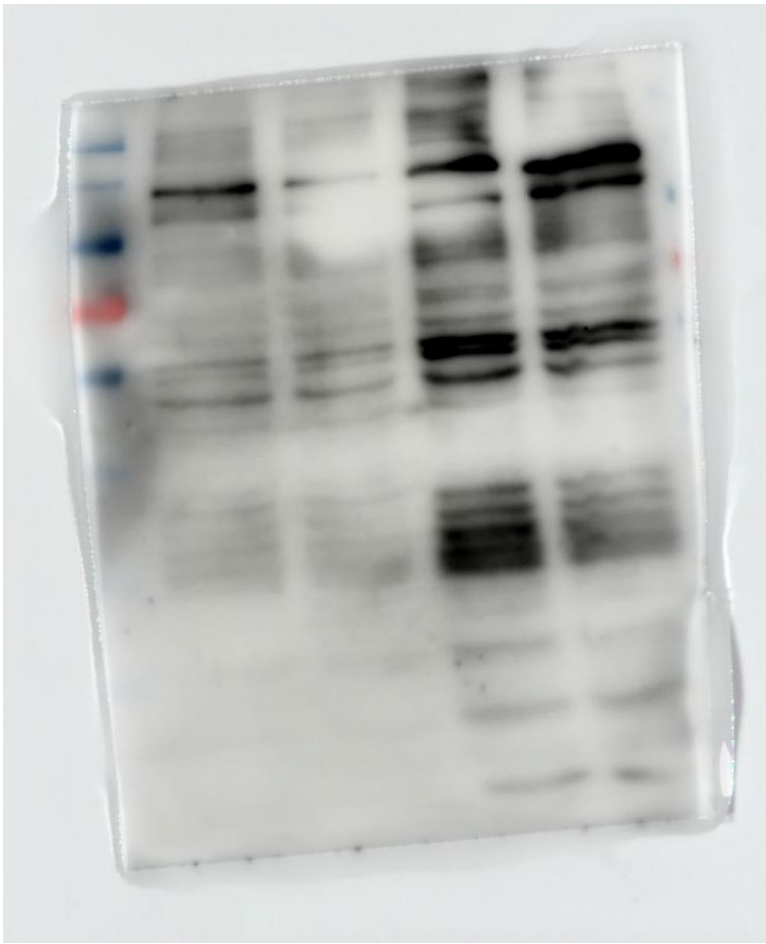

$\beta$ -ACTIN

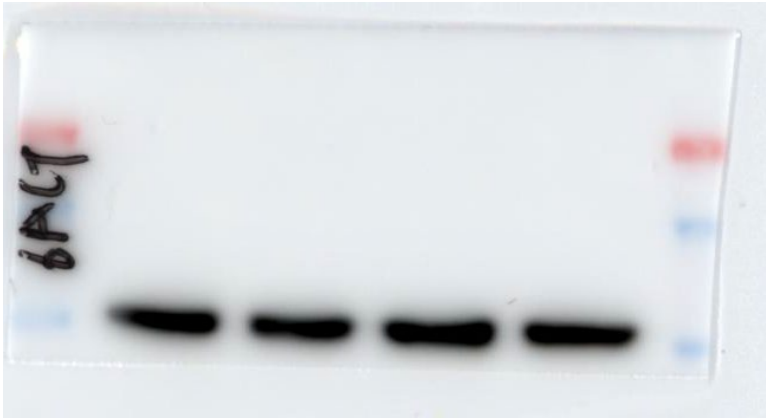

Supplement: Supplementary file 1 [file Image1.pdf]
