# Supplementary material for: Hysterectomy accelerates sarcopenia risk in US women and mouse models
Source: Front Endocrinol (Lausanne). 2026 Jul 14;17:1859421. doi: 10.3389/fendo.2026.1859421 (PMC13407118; doi:10.3389/fendo.2026.1859421)
Supplement: Supplementary file 2 [file Table1.docx]

Supplementary Table S1：Primers Used for RT-qPCR Analysis.

| Gene name | Forward (5′→3′) | Reverse (5′→3′) |
| --- | --- | --- |
| Murf-1 | ACCTGCTGGTGGAAAACATC | AGGAGCAAGTAGGCACCTCA |
| Atrogin-1 | CAGTGAGCCCTGCCATAACA | TCCCAAGTATGGAGCAGGGA |
| Foxo1 | CCACCACAGCGGACTTGAGTAA | AGCAATGGAACAGGAGCAAGG |
| Gpx4 | GAGATCAAAGAGTTCGCCGC | GGAGAGACGGTGTCCAAACT |
| Acsl4 | TATGGGCTGACAGAATCATG | CAACTCTTCCAGTAGTGTAG |
| β-Actin | GTGACGTTGACATCCGTAAAGA | GCCGGACTCATCGTACTCC |
